# Evaluation of effects of a novel probiotic feed supplement on the quality of broiler meat

**DOI:** 10.14202/vetworld.2019.1775-1778

**Published:** 2019-11-13

**Authors:** Ujang Suryadi, Yudhi Ratna Nugraheni, Anang Febri Prasetyo, Aan Awaludin

**Affiliations:** 1 Department of Animal Science, Politeknik Negeri Jember, Mastrip Street PO. BOX 164, Jember, Indonesia; 2 Department of Parasitology, Faculty of Veterinary Medicine, Universitas Gadjah Mada, Jl. Fauna No. 2, Karangmalang, Yogyakarta, Indonesia

**Keywords:** broiler, chicken, meat, microorganism, probiotic

## Abstract

**Background and Aim:**

A local microorganism-based probiotic has been developed as an alternative to using antibiotic growth promoter and its effect on broiler meat quality has been studied, when supplemented with poultry feed at different concentrations. This study aimed to understand the effect of local microorganism-based probiotic sourced from cattle rumen and chicken intestine and added as feed supplement at different concentrations on broiler meat quality.

**Materials and Methods:**

The local microorganism-based probiotic made from cattle rumen and chicken intestine contained *Lactobacillus* spp., *Bifidobacterium* spp., *Streptococcus* spp., and *Bacillus* spp. The experiments followed a completely randomized design. Treatments in the study were: P0, i.e., control (without probiotic), P1 (probiotic administered at 5 ml/kg feed), P2 (probiotic administered at 10 ml/kg feed), and P3 (probiotic administered at 15 ml/kg feed). Each treatment was repeated 5 times. Parameters examined in this study were pH, meat tenderness, fat content, and meat protein content.

**Results:**

Based on a total of 200 chickens, the percentage of meat protein content in treatments P1, P2, and P3 showed an increase of 19.34%, 19.42%, and 19.64%, respectively, when compared with P0 that showed a protein content increase of 19.14%. The fat content of meat for P1, P2, and P3 was 21.54%, 21.46%, and 21.30%, respectively, which was less than the value for P0 (21.69%). The treatments did not significantly affect pH or meat tenderness when compared with the control. The usage of this novel probiotic as a feed supplement resulted in an increase in meat protein content and a decrease in fat content.

**Conclusion:**

This study indicates that using the local microorganism-based probiotic sourced from cattle rumen and chicken intestine to supplement poultry feed did not have a significantly different effect (p>0.05) on meat pH; however, it had a significantly different (p<0.05) on protein and fat content of broiler meat.

## Introduction

The use of antibiotics in poultry plays a key role in curbing infectious diseases and stimulating poultry growth [1]. Antibiotics should be used with caution and must be regulated by an external party. Proper management of antibiotics is a good way to limit the negative effects of antibiotic abuse and ensure the safety of food derived from animals and the environment they live in [2]. Prohibition of antibiotic growth promoters (AGPs) encouraged breeders to search for an alternative to AGP that has similar advantageous effects such as optimizing animal performance, increasing nutrient availability, and improving feed conversion and/or growth [3].

Probiotics have the ability to change the intestinal microbial environment, improve intestinal immunity, increase resistance to disease, decrease pathogenic infections and symptoms of diseases, and improve health [4,5]. FAO/WHO portrays probiotics as microorganisms which, when given in effective doses, improves the balance of microorganism communities in the host’s digestive system, which has many medical benefits. Probiotics may contain one or more types of microorganisms and can be administered both individually and in combination with other additives in feed or water [6,7]. However, most of the current commercial products contain more than one microbial species, and some products may even contain viable yeast and other types of fungus [6]. Some of the commonly used probiotic microorganisms are *Lactobacillus rhamnosus*, *Lactobacillus reuteri*, and *Bifidobacterium* spp., certain strains of *Lactobacillus casei*, *Lactobacillus acidophilus*, *Bacillus coagulans*, and *Escherichia coli* strain Nissle 1927, certain enterococci, especially *Enterococcus faecium* SF68, and the yeast *Saccharomyces boulardii* [8]. Various bacteria (*Bacillus*, *Bifidobacterium*, *Enterococcus*, *Lactobacillus*, *Streptococcus*, and *Lactobacillus* spp.) and in some cases, yeast (*Saccharomyces* spp.) have been tested for their effectiveness as probiotics in poultry [9]. Local probiotics comprising rumen bacteria combined with chicken intestinal bacteria can be produced using simple technologies so that breeders can make these probiotics themselves. Probiotics in poultry have been demonstrated to be useful in improving pH, color, water holding capacity, chemical composition, fatty acid profile, and oxidative strength. According to Popova [10], some bacterial genera that can be used as probiotics are as follows: *Lactobacillus*, *Bacillus*, *Bifidobacterium*, *Enterococcus*, and *Escherichia*. Probiotics play a key role in nutritional retention and digestion by increasing digestive enzyme activities and by improving the solubility of nutrients [10]. Supplementing the feed with probiotics results in probiotic microorganisms breaking down the nutritional content of the feed into smaller components that are easily absorbed by the body, thereby increasing growth, body weight, and efficiency of feed absorption in broiler poultry given probiotics. Some probiotics do this by producing digestive enzymes such as amylase, protease, and lipase, which can increase the digestive enzyme concentration in the host’s digestive tract, and increase nutrient reshuffle [11].

The provision of local microorganism-based probiotics from rumen bacteria combined with chicken intestinal bacteria resulted in the alteration of pH, water binding capacity, tenderness of meat, and shrinkage of broiler meat during cooking.

This study aimed to determine the effect of the administration of a novel probiotic as a feed supplement on the quality of broiler meat. The examined parameters included meat fat content, protein content, and pH.

## Materials and Methods

### Ethical approval

All procedures performed in this study involving chickens were in accordance with the ethical standards of the Politeknik Negeri Jember, where the study was conducted.

### Materials

This study used 200 individuals of Day-Old Cobb broiler chicken strains that were reared for 35 days. The feed used in this study was primarily the BR1 “Patriot Feed,” with a crude protein content of 20%, a maximum of 5% crude fiber, a maximum crude fat of 6%, calcium content of 0.9-1.1%, and phosphorus content of 0.7-0.9%. The composition of bacteria used in the probiotic for this research was as follows: *Bifidobacterium* bacteria 3.5×10^7^ colony-forming units (CFU)/ml, *Bacillus* 0.9×10^6^ CFU/ml, *Streptococcus* 1.06×10^6^ CFU/ml, and *Lactobacillus* 12.5×10^7^ CFU/ml. Five-month-old domestic chickens and 2-year-old cattle were used as the source of microorganisms. The novel probiotic supplement was given to all chickens except those in the control group from day 1 to day 35.

### Methods

The following is the method to make probiotic using local microorganisms sourced from cattle rumen and chicken intestine: One kilogram of potatoes was boiled in 5 L of water. Ten liters of this boiled solution was then mixed with 1 kg of molasses and 0.5 kg of shrimp paste and boiled. The solution was poured into a bucket filled with 5 kg of corn bran, mixed until smooth, then left to stand overnight in the bucket. Juice was extracted from 1 kg of pineapples, left to stand for 1 h, and then mixed with 1 kg of cattle rumen and 1 kg of chicken intestines. The solution was left to stand for 7 days. Once the excess liquid is filtered out, the probiotics will be present in the filtrate.

The study method used was based on a completely randomized design, with four treatments and five replicates of each treatment. There were 50 chickens in each treatment. The treatments were as follows:

P0 = Treatment without novel probiotic feed supplementation (control)

P1 = Novel probiotic feed supplemented at a concentration of 5 ml/kg feed

P2 = Novel probiotic feed supplemented at a concentration of 10 ml/kg feed

P3 = Novel probiotic feed supplemented at a concentration of 15 ml/kg feed

### Statistical analysis

The parameters observed in this study were meat fat content, protein content, meat tenderness, and pH. The data of the study results were analyzed in SPSS 17 (IBM, USA) using one-way analysis of variance (ANOVA) and Tukey’s honestly significant difference test, according to LeBlanc [12].

## Results and Discussion

The chemical qualities of broiler meat with the addition (supplementation) of probiotics made using local microorganisms sourced from cattle rumen and chicken intestine are shown in Table-1.

**Table-1:** Percentage change in parameter according to treatment for each group.

Treatment	Parameter (mean)			

	Protein (%)	Fat (%)	pH	Meat tenderness
P0	19.14^a^	21.69^a^	6	15.4
P1	19.34^b^	21.54^b^	6	15.8
P2	19.42^b^	21.46^b^	6	15.1
P3	19.64^c^	21.30^c^	6	15.2

Superscript letters indicate that the mean difference is significant at 0.01 level (p<0.01)

The results of the statistical analyses showed that broilers fed without the addition of the novel probiotic (P0) had a significantly higher fat content (p<0.01) than did broilers provided with feed supplemented with the probiotic in treatments P1, P2, and P3. The fat content of broiler meat varied between treatments supplemented with probiotics; the administration of the probiotic at a concentration of 15 ml/kg (P3) resulted in significantly less fat (p<0.01) than did the administration of both a lower (5 ml/kg [P1]) and a higher (10 ml/kg [P2]) probiotic content, while the effects of administration of P1 and P2 did not vary significantly (p>0.05).

The previous study reported that given novel probiotics feed supplement was not significantly different (p>0.05) compared to broilers fed BR1-commercial feed between the use of probiotic levels not significantly different (p>0.05). This shows that local microorganism probiotics can be used as an alternative to AGP without reducing the nutritional quality of broiler meat [13].

Lipase and esterase enzymes are produced by probiotic bacteria and can break ester bonds that link glycerol to fatty acids, preventing the formation of triglycerides. This results in decreased absorption of triglycerides into plasma, thereby reducing the fat content of meat. For this reason, broilers supplemented with probiotics made using microorganisms from cattle rumen, and chicken intestines produced less fatty meat. According to Mahdavi *et al*. [14], probiotics are capable of integrating esterase enzymes with lipase enzymes by breaking up ester bonds, which link glycerol and fatty acids through esterification. This reduces the quantity of triglycerides in intestines, which ultimately reduces triglyceride absorption in the plasma. Lipids are stored in adipose and muscle tissues in the form of triglycerides, so when the absorption of triglycerides decreases, the fat content of meat also decreases, resulting in less fatty meat.

The fat content of meat of broilers fed with the highest concentration of probiotics (P3) was significantly lower than that in meat of broilers under P1 and P2 treatments and was caused by probiotic supplementation of more *Lactobacillus* and *Bacillus* spp. According to Kalavathy *et al*. [15], *Lactobacillus* culture in probiotics could function to reduce fat content in muscles and liver. *Bacillus* spp. can synthesize lipase and protease enzymes, which help in the process of digestion and absorption of nutrients. Therefore, an increase in the activity of lipase enzymes could reduce fat in the digestive tract of poultry [16]. The reduction of fat would decrease the absorption of triglycerides in the blood, resulting in a decrease in the fat content of meat. The effect of local microorganism-based probiotic made from cattle rumen and chicken intestine on the fat content of broiler meat is represented in Tabel-1

The results of the protein content of broiler meat that had been supplemented with probiotics are shown in Table-1. The results of the ANOVA indicate that there is a significant effect (p<0.01) of probiotic administration on meat protein content. In chickens not supplemented with probiotics (P0), the meat protein content was significantly lower (p<0.01) than feed supplemented with the novel probiotic supplement (P1, P2, and P3). Treatments supplemented with probiotics of different concentrations showed that the administration of probiotics with a concentration of 15 ml/kg (P3) contained significantly more protein (p<0.01) than giving probiotics at a concentration of 5 ml/kg (P1) and 10 ml/kg (P2). Protein content in treatments P1 and P2 was not significantly different (p>0.05) from each other.

The high protein content of broilers supplemented with the novel probiotics is potentially due to the presence of lactic acid bacteria. The bacteria are able to survive in the digestive tract, and bind to the intestinal walls, producing digestive enzymes such as proteases. Widiyaningsih suggests that probiotics containing lactic acid bacteria can produce digestive enzymes that break down chemical bonds in nutrients, making molecules smaller and easier to absorb, resulting in accelerated absorption of these nutrients, including protein [17]. The previous study by Wang stated that probiotics could increase chicken meat growth and increase microbiota diversity in the intestines [18]. According to Fooks and Gibson [11], probiotics produced digestive enzymes such as amylase, protease, and lipase, which could accelerate nutrient reshuffle, where specific enzymes help with the fermentation of carbohydrates.

Feed supplemented with the highest concentration of probiotics yielded meat with the most protein. This is thought to be due to the presence of more lactic acid bacteria that produce more digestive enzymes, thereby optimizing protein absorption. This is in accordance with what was found in a previous study [19] that increased protein absorption results in higher protein content in meat. The availability of protein in simpler forms strongly indicates increased meat protein synthesis that leads to increased protein deposition, which in this study was manifested in increased meat protein content.

The average pH content of broilers was the same across both control and treatments with probiotic supplements. The same pH value in broiler meat is thought to be due to chickens in this study having a healthy body, and their condition remained healthy even after being supplemented with probiotics. In healthy chickens, metabolic processes such as glycolysis would continue producing glycogen, so the pH would not significantly change. Anaerobic glycolysis metabolism generates fatty acid accumulation in the muscle, and it can alter the pH. Chicken health was instead improved by the probiotic working to inhibit growth of and kill pathogenic bacteria in chicken intestines such as *Lactobacillus*, *Bifidobacterium*, *Salmonella*, *Campylobacter*, *E. coli*, and *Clostridium*. In young chicks, the dominant species present in the small intestine and ceca is Lactobacilli, with Bifidobacteria becoming more common in the ceca with age. Clostridium was detected in some segments of the small intestine in young chicks, according to Amit-Romach *et al*. [20] This is related to the finding [8] that bacteria such as *Bifidobacterium*, *Lactobacilli*, *S. boulardii*, and *B. coagulans* can strengthen the immune system, increase metabolism, and restore the balance of the intestinal flora. Given the improved microbial balance in broiler gut, broiler health would also improve. According to Raharjo *et al*. [21], healthy chickens after slaughter still had a lot of glycogen in their muscles so that as glycolysis continues, the pH does not drop too much. The tenderness of meat can be influenced by heat. The values of meat tenderness and pH did not significantly differ between treatments and the control, which is in line with previous findings [22]. Other research has also reported that probiotics do not influence pH and meat tenderness, but probiotics can still be used as an alternative to antibiotics for poultry [23].

## Conclusion

Supplementation of feed with a novel probiotic comprising microorganisms from cattle rumen and chicken intestine had a significant effect (p<0.01) on increasing the protein content and reducing the fat content but had no significant effect (p>0.05) on broiler meat pH and meat tenderness. Administration of the probiotic at the rate of 15 ml/kg of feed gave the lowest fatty content and the highest broiler protein content.

## Authors’ Contributions

US designed the study, US and YRN made probiotic composition, AA and AFP examined the sample in the laboratory. All authors drafted and revised the manuscript. All authors read and approved the final manuscript.
